# Propolin C Inhibited Migration and Invasion via Suppression of EGFR-Mediated Epithelial-to-Mesenchymal Transition in Human Lung Cancer Cells

**DOI:** 10.1155/2018/7202548

**Published:** 2018-02-25

**Authors:** Jih-Tung Pai, Yi-Chin Lee, Si-Ying Chen, Yann-Lii Leu, Meng-Shih Weng

**Affiliations:** ^1^Division of Hematology and Oncology, Taoyuan General Hospital, Ministry of Health and Welfare, Taoyuan City, Taiwan; ^2^Department of Nutritional Science, Fu Jen Catholic University, New Taipei City, Taiwan; ^3^Graduate Institute of Natural Products, College of Medicine, Chang Gung University, Taoyuan 33302, Taiwan; ^4^Center for Traditional Chinese Medicine, Chang Gung Memorial Hospital, Taoyuan City, Taiwan

## Abstract

Controlling lung cancer cell migration and invasion via epithelial-to-mesenchymal transition (EMT) through the regulation of epidermal growth factor receptor (EGFR) signaling pathway has been demonstrated. Searching biological active phytochemicals to repress EGFR-regulated EMT might prevent lung cancer progression. Propolis has been used as folk medicine in many countries and possesses anti-inflammatory, antioxidant, and anticancer activities. In this study, the antimigration and anti-invasion activities of propolin C, a c-prenylflavanone from Taiwanese propolis, were investigated on EGFR-regulated EMT signaling pathway. Cell migration and invasion activities were dose-dependently suppressed by noncytotoxic concentration of propolin C. Downregulations of vimentin and snail as well as upregulation of E-cadherin expressions were through the inhibition of EGFR-mediated phosphatidylinositol-3-kinase/protein kinase B (PI3K/Akt) and extracellular signal-regulated kinase (ERK) signaling pathway in propolin C-treated cells. In addition, EGF-induced migration and invasion were suppressed by propolin C-treated A549 lung cancer cells. No significant differences in E-cadherin expression were observed in EGF-stimulated cells. Interestingly, EGF-induced expressions of vimentin, snail, and slug were suppressed through the inhibition of PI3K/Akt and ERK signaling pathway in propolin C-treated cells. Inhibition of cell migration and invasion by propolin C was through the inhibition of EGF/EGFR-mediated signaling pathway, followed by EMT suppression in lung cancer.

## 1. Introduction

Propolis is a resinous material collected from buds and exudates of plants to build and defend the hive by honeybee. Propolis has been used as folk medicine for a long time in many countries. Numerous studies have indicated the biological activities of propolis, such as antimicrobial, antiviral, anti-inflammatory, antioxidant, and antitumor activities [1–4]. The biological components of propolis are highly diverse due to different plant species of propolis source. According to plant origins and chemical compositions, propolis can be divided into six categories in the world [5]. The main bioactive compounds of propolis from Europe and China are flavonoids and phenolic acids [6, 7]. Otherwise, Brazilian propolis mainly contains terpenoids and prenylated derivates of* p*-coumaric acids [8, 9]. The c-prenylflavanones are specific active constitutes of propolis from east Pacific regions, such as Taiwan and Okinawa [10, 11]. Eight prenylflavanones of Taiwanese propolis, propolin A to H, have been identified and originate from the surface resinous materials of* Macaranga tanarius* L. fruits [12, 13]. The anticancer activities of propolins have been characterized in lung cancer, melanoma, and glioma cell models [14–18]; however, the anti-invasion and antimigration activities of these components are still unclear.

Lung cancer is the leading cause of cancer death worldwide. Histologically, non-small cell lung cancer (NSCLC) accounts for about 80% of lung cancer [19]. Aberrant activation of epidermal growth factor receptor (EGFR) signaling pathway has been identified to advance lung cancer tumorigenesis and results in the increase of patients mortality [20, 21]. EGFR is a receptor tyrosine kinase of ErbB family including EGFR (ErbB1), HER2 (Neu, ErbB2), HER3 (ErbB3), and HER4 (ErbB4). EGFR activation is induced by ligand and epidermal growth factor (EGF) binding and leads to dimerization, autophosphorylation, and activation of downstream signaling pathways. PI3K/Akt and MEK/ERK are the dominant two downstream signaling pathways of EGFR and are involved in EGFR-mediated cell proliferation, differentiation, and metastasis [22]. Dysregulation of EGF/EGFR signaling pathway is a well-known critical factor of lung cancer tumorigenesis and for the poor prognosis of this cancer [21]. Therefore, targeting EGF/EGFR signaling pathway to attenuate cancer cell tumorigenesis is the strategy for preventing and/or improving lung cancer patient prognosis.

Epithelial-to-mesenchymal transition (EMT) is a critical mechanism to regulate embryonic development, wound healing, and cancer cell metastasis [23]. EMT is a process in which cells lose their epithelial properties and convert to mesenchymal characteristics. During EMT development, the cell-cell cohesive ability is lost, and the migration capacity emerges [24]. In this process, molecularly, the epithelial-type molecules, such as E-cadherin and cytokeratin intermediate filament proteins, are downregulated; and, in contrast, the mesenchymal-type markers, such as N-cadherin, vimentin, and EMT-associated transcription factors, are upregulated [25]. Cumulative evidences demonstrate that EMT is a critical mechanism in tumor metastasis and impact on prognosis patient [26]. Induction of EMT by various growth factors, such as hepatocyte growth factor, transforming growth factor, and EGF, has been clarified in many cancer cell models [27, 28]. EGF-induced EMT has been demonstrated via ERK-mediated downregulation of E-cadherin and upregulation of vimentin and snail [29]. Furthermore, suppression of PI3K/Akt signaling pathway by quercetin has been examined in EGF-induced EMT in prostate cancer. EGF-induced expression of mesenchymal-like molecules, such as N-cadherin, vimentin, snail, and slug, is repressed by quercetin. Meanwhile, decreasing the expression of E-cadherin by EGF is reversed in quercetin-treated prostate cells [30].

Although the biological active components [12] and the anticancer properties of Taiwanese propolis have been examined [14–18], many biological functions and molecular mechanisms of Taiwanese propolis are still mystery, especially in EGF/EGFR-regulated tumorigenesis. In this study, the antimigration and anti-invasion properties of propolin C, a biological active compound of Taiwanese propolis, were examined in EGF/EGFR-regulated EMT in lung cancer. The results implicated that propolin C could be developed as a potential preventive agent for lung cancer metastasis.

## 2. Materials and Methods

### 2.1. Purification of Propolin C

**Figure 1:**
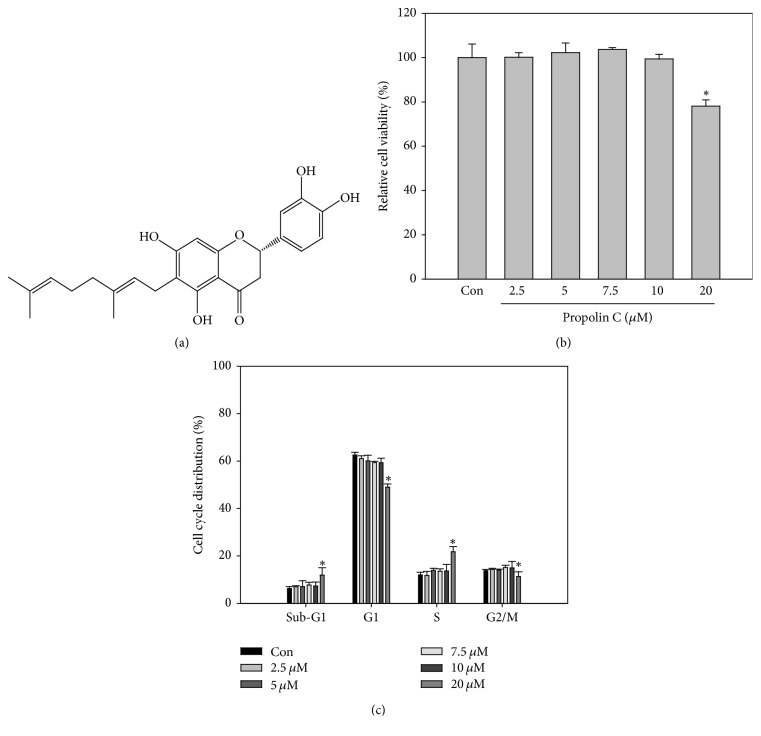
*Inhibitory effects of propolin C on cell viability of human lung carcinoma cancer cell line*. (a) Chemical structure of propolin C. (b) HCC827 cells were cultured in 96-well plates and treated with propolin C (2.5, 5, 7.5, 10, and 20 *μ*M) for 24 h. After incubation, cell viability and (c) cell cycle distribution were detected by MTT assay and flow cytometry with PI labeling, respectively. Data were the mean ± SD of triplicate samples. ^*∗*^*p* < 0.05 compared with control cells.

Taiwanese propolis was purchased from Hualian, Taiwan. A voucher specimen (CGU-PE-1) was deposited in the herbarium of Chang Gung University, Taoyuan, Taiwan. Taiwanese propolis (985 g) was extracted with ethanol (5 L × 6) at room temperature. The filtered ethanol extracts were collected and concentrated to gain brown syrup (902.2 g). The brown syrup of ethanol extract was subjected to column chromatography (column: 14 cm i.d. × 75 cm) over silica gel (SiliaFlash G60, SiliCycle) and eluted with CH_2_Cl_2_ and EtOAc step gradients to acquire seven fractions. The second fraction (CH_2_CL_2_ : EtOAc = 9 : 1, 6 L) was concentrated and purified by recrystallization to obtain propolin C (22.206 g). The structure of propolin C was identified by comparison of the spectral data with literature values (Figure 1(a)) [14].

### 2.2. Chemicals, Reagents, and Antibodies

Anti-p-EGFR, anti-EGFR, anti-p-ERK, anti-ERK, anti-p-Akt, anti-slug, and anti-snail antibodies were obtained from Cell Signaling Technology (Beverly, MA, USA). Anti-Akt and anti-vimentin antibodies were acquired from GeneTex, Inc. (Irvine, CA, USA). Anti-E-cadherin antibody was purchased from BD Biosciences, Inc. (San Jose, CA, USA). Anti-*β*-actin antibody was purchased from Santa Cruz Biotechnology (Santa Cruz, CA, USA).

### 2.3. Cell Culture and Cell Viability Assays

The A549 and HCC827 lung cancer cell lines were purchased from the American Type Culture Collection (Manassas, VA, USA). Both of the cell lines were maintained in 5% fetal bovine serum-containing RPMI-1640 (HyClone Laboratories, Logan, UT, USA) and cultured at 37°C in 5% CO_2_ atmosphere. Cells (1 × 10^4^/well) were seeded in 96-well plates for 24 h and then incubated with propolin C (0, 2.5, 5.0, 7.5, 10, and 20 *μ*M) for 24 h. After treatment, cell viability was examined by MTT assay.

### 2.4. Cell Cycle Analyses

Cell cycle analysis was performed as described previously [31]. Briefly, cells were seeded and synchronized. After synchronization, propolin C-containing 5% fetal bovine serum medium was incubated for 24 h. Cells were harvested and stained with propidium iodide (50 *μ*g/mL, Sigma-Aldrich, St. Louis, MO, USA), and FACScan laser flow cytometer analysis system (Beckman Coulter, Fullerton, CA) was used to detect cell cycle distribution.

### 2.5. *In Vitro* Wound Closure

A549 and HCC827 cells (1 × 10^5^ cells/well) were plated in 6-well plates for 24 h. Cells wounded by scratched with a pipette tip, incubated with or without propolin C (0, 2.5, 5.0, 7.5 and 10 *μ*M)-containing 0.5% FBS RPMI medium for 24 h. Cells were photographed using a phase-contrast microscope (×200), as the descriptions in Kao et al. [32].

### 2.6. *In Vitro* Invasion and Migration Assays

*In vitro* invasion and migration assays were measured by modified protocols from Kao et al. [32]. Briefly, HCC827 and A549 cells were treated with serial concentrations of propolin C (0, 2.5, 5.0, 7.5, and 10 *μ*M) for 24 h and cells were collected to be plated on Boyden chamber (BD Biosciences, Bedford, MA, USA) at cell density of 1 × 10^5^ cells/well in serum-free medium for 24 h incubation. For* in vitro* invasion assay, 8 *μ*m pore polycarbonate filters were coated with 10 *μ*l Matrigel (25 mg/mL; BD Biosciences, Bedford, MA, USA) and the lower chamber was contained in 5% FBS-containing RPMI-1640 medium. The invaded cells were fixed with methanol and stained with 0.1% crystal violet. Cell numbers were counted under a light microscope.* In vitro* migration assay, 8 *μ*m pore polycarbonate filters were not coated with Matrigel and experimental processes were the same as* in vitro* invasion assay. Triplicate samples were conducted, and data were expressed as average cell number.

### 2.7. Western Blot Analyses

Western blot analyses were performed as described previously [31]. Briefly, cell lysates were prepared and then quantitated, electrophoresed via sodium dodecyl sulfate-polyacrylamide gel electrophoresis (SDS-PAGE), and then transferred to Immobilon polyvinylidene difluoride membranes (Millipore Co., Billerica, MA, USA). After transfer, the membranes were blocked and incubated with the indicated antibodies. The signals were detected by chemiluminescence (ECL Kit, Amersham Pharmacia Biotech, IL, USA). The intensities of protein expression were then quantitated by a BioSpectrum Imaging System ChemiDoc-It2 810 (UVP, LLC, CA, USA). The expression of *β*-actin was used as the internal control.

### 2.8. Statistical Analyses

The results were expressed as the mean ± SD calculated from at least three independent determinations. One-way analysis of variance (ANOVA) tests were used to compare individual experiment with the control value. ANOVA with Duncan's post hoc test in SAS statistical software was used for statistical analysis for the comparisons with different treatment groups. A *p* < 0.05 was considered as a significant difference.

## 3. Results

### 3.1. The Antiproliferative Effects of Propolin C in HCC827 Lung Cancer Cells

To evaluate the antiproliferative activity of propolin C on EGFR-mutated lung cancer cells, HCC827 cells were incubated with serial dosages of propolin C for 24 h and cell viability was then detected. As shown in Figure 1, there was no significant difference in cell viability after treatment with 10 *μ*M of propolin C. However, cell viability decreased about 20% after treatment with 20 *μ*M of propolin C (Figure 1(b)). Furthermore, treatment with 10 *μ*M of propolin C did not affect cell cycle distribution. The sub-G1 and S phase cell accumulations were only observed in cells treated with 20 *μ*M of propolin C (Figure 1(c)). According to these results, concentration of propolin C with no cytotoxicity effect for further examination was chosen.

### 3.2. Effects of Propolin C on Cell Migration and Invasion in HCC827 Lung Cancer Cells

**Figure 2:**
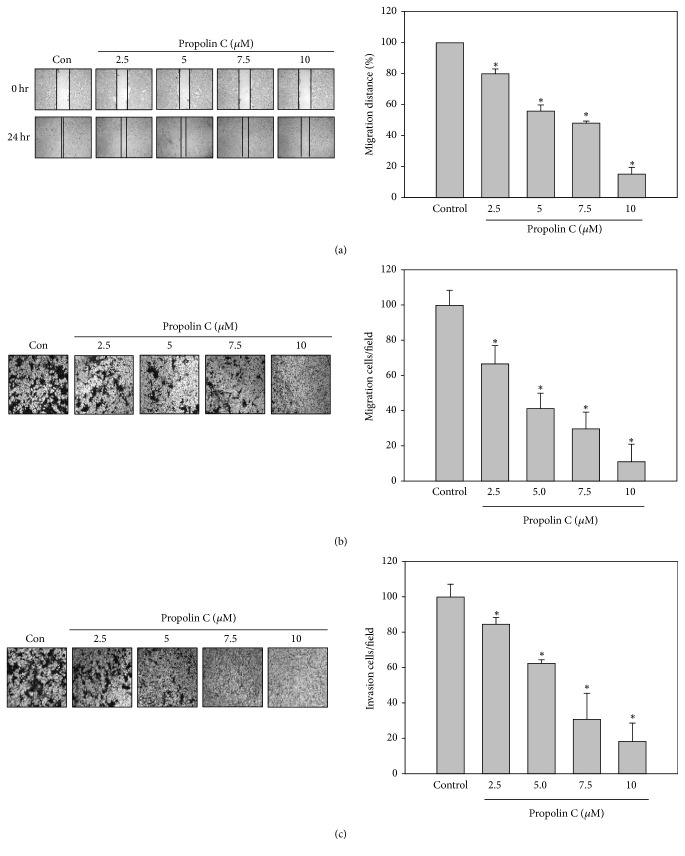
*Inhibitory effects of in vitro migration and invasion by propolin C in HCC827 lung cancer cells*. HCC827 cells were incubated with serial dosages of propolin C (2.5, 5, 7.5, and 10 *μ*M) for 24 h and (a)* in vitro* wound healing, (b) Transwell migration, and (c) invasion analyses were performed as described in Materials and Methods. Data were represented as the mean ± SD of triplicate samples. Significant difference was observed from the control group (^*∗*^*p* < 0.05).

To examine the antimigration and anti-invasion activities of propolin C, HCC827 cells were incubated with serial dosages of propolin C (0, 2.5, 5, 7.5, and 10 *μ*M) for 24 h and* in vitro* migration and invasion assay was assessed subsequently. The results revealed that propolin C inhibited cell migration in a dose-dependent matter by wound healing and* in vitro* migration analyses (Figures 2(a) and 2(b)). Meanwhile, suppression of cell invasion was also observed in propolin C-treated cells in a dose-dependent manner (Figure 2(c)).

### 3.3. The Role of PI3K/Akt and ERK Signaling Pathway in Propolin C-Regulated EMT

**Figure 3:**
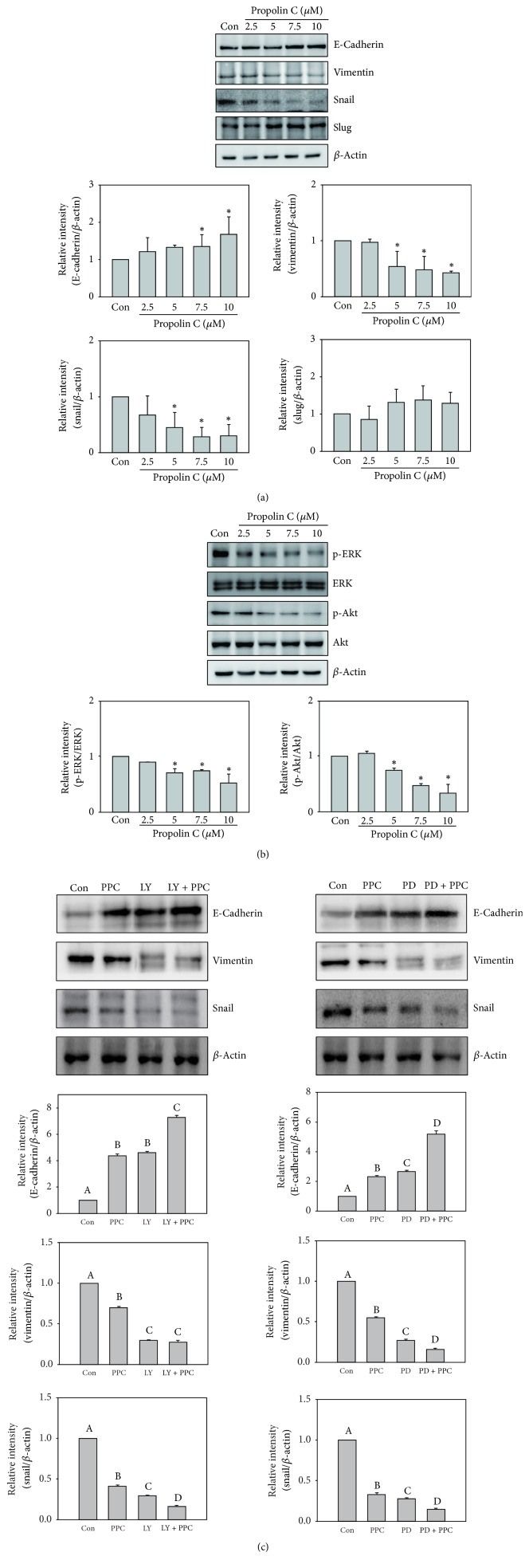
*Effects of propolin C on PI3K/Akt and ERK-mediated EMT marker expressions in HCC827 lung cancer cells*. HCC827 cells were treated with propolin C (2.5, 5, 7.5, and 10 *μ*M) for 24 h. After treatment, the expressions of (a) E-cadherin, vimentin, slug, snail, and *β*-actin and (b) phospho-ERK, ERK, phospho-Akt, and Akt were analyzed by Western blot as described in Materials and Methods. Significant difference was observed from the control group (^*∗*^*p* < 0.05). (c) HCC827 cells were pretreated with LY294002 (LY, 10 *μ*M) or PD98059 (PD, 10 *μ*M) for 30 min and then incubated with or without propolin C (PPC, 10 *μ*M) for 24 h. After incubation, cells were harvested and Western blot analyses were used to detect E-cadherin, vimentin, snail, and *β*-actin expressions. Data were shown as mean ± SD (*n* = 3). Different uppercase letters (A−D) indicate statistical differences among group (*p* < 0.05), and the same letter showed no difference (*p* > 0.05).

To understand antimigration and anti-invasion effect of propolin C on EMT regulation, HCC827 cells were incubated with serial dosages of propolin C (0, 2.5, 5, 7.5, and 10 *μ*M) for 24 h and EMT molecule expressions were detected by Western blot. The expression of epithelial-like cell marker, E-cadherin, was upregulated with the treatment of 7.5 *μ*M propolin C, and the expression of mesenchymal-like cell marker, vimentin, was inhibited in a dose-dependent matter. Meanwhile, transcription factor, snail, but not slug, was dramatically decreased with propolin C treatment (Figure 3(a)).

EMT has been well known to be regulated by PI3K/Akt and ERK signaling pathways. To investigate suppression of EMT by propolin C, the expressions of phosphorylated Akt and ERK were evaluated. As shown in Figure 3(b), the expressions of phospho-ERK and phospho-Akt were dose-dependently inhibited by propolin C. Furthermore, protein expression of E-cadherin in the treatment with PI3K/Akt inhibitor (LY294002) or ERK inhibitor (PD98059) alone was upregulated, resulting in the inhibition of vimentin and snail (Figure 3(c)). Interestingly, treatment with LY294002 or PD98059 enhanced propolin C-induced E-cadherin expression. In addition, the inhibitions of vimentin and snail expression were also enhanced in combination treatment, compared with propolin C treatment alone (Figure 3(c)).

### 3.4. The Effects of Propolin C-Regulated EMT on EGFR Signaling Downregulation

**Figure 4:**
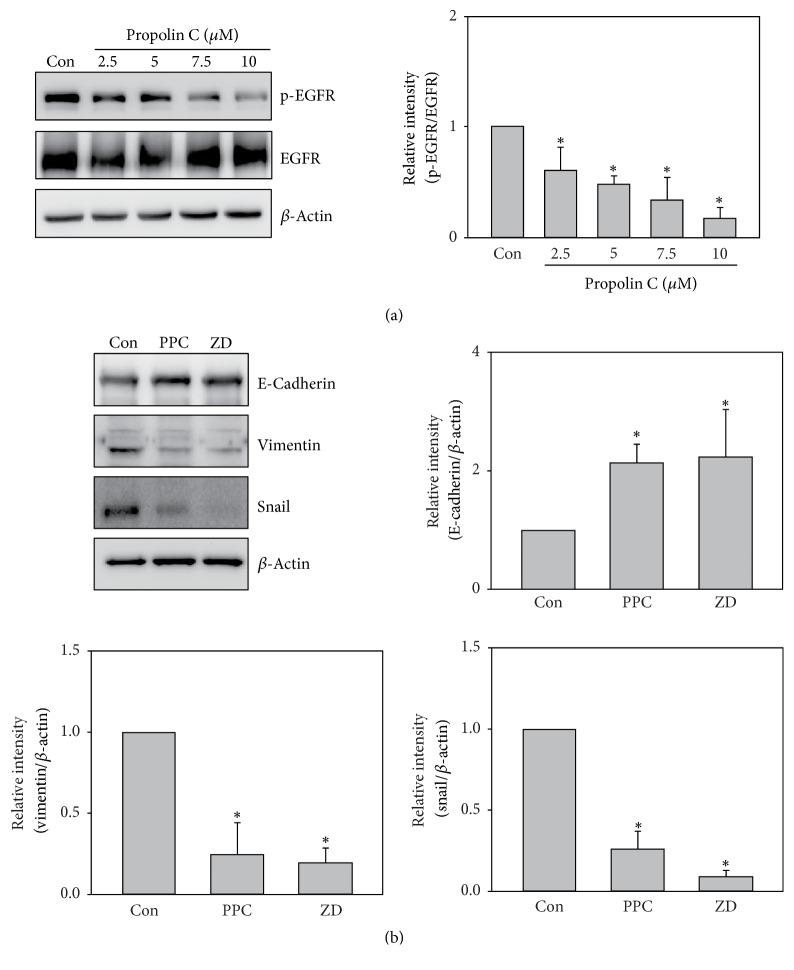
*Effects of propolin C on EGFR-mediated EMT marker expressions in HCC827 lung cancer cells*. HCC827 cells were incubated with (a) propolin C (2.5, 5, 7.5, and 10 *μ*M) or (b) 10 *μ*M of propolin C (PPC) and 1 nM of ZD1839 (ZD) for 24 h. After incubation, cell lysates were harvested and Western blot analyses were performed to detect the expressions of E-cadherin, vimentin, snail, and *β*-actin. Data represented at least three independent experiments. Significant difference was observed from the control group (^*∗*^*p* < 0.05).

EGFR-mediated PI3K/Akt and ERK signaling pathways are important in EMT regulation. Suppression of PI3K/Akt and ERK signaling pathway by propolin C inhibited EMT in propolin C-treated HCC827 cells (Figure 3). To measure the target of propolin C treatment, phosphorylation of EGFR was estimated in propolin C-treated HCC827 cells. The results revealed that the expression of phospho-EGFR was decreased in a dose-dependent manner (Figure 4(a)). To further assess the role of EGFR in propolin C-regulated EMT, clinical EGFR inhibitor, ZD1839, was used. The expression of E-cadherin was increased, whereas the expressions of vimentin and snail were decreased in ZD1839-treated cells (Figure 4(b)). Meanwhile, the expression of EMT molecular prolife in propolin C-treated cells was similar to ZD-treated cells.

### 3.5. The Suppression of EGF-Induced Cell Migration and Invasion by Propolin C in A549 Lung Cancer Cells

**Figure 5:**
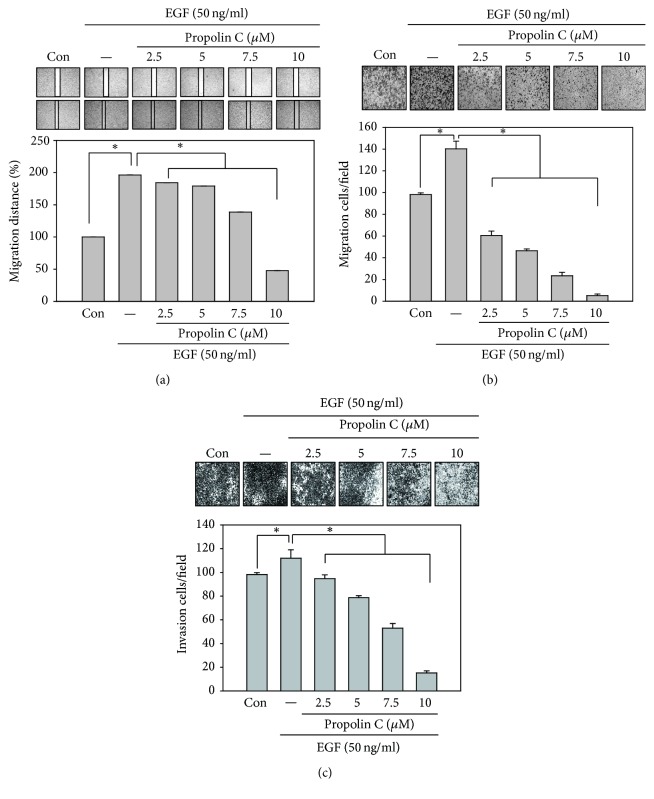
*Effects of propolin C on EGF-induced migration and invasion in A549 lung cancer cells*. A549 cells (1 × 10^4^/well) were pretreated with propolin C (2.5, 5, 7.5, and 10 *μ*M) for 30 min and then stimulated with 50 ng/mL of EGF for 24 h. After treatment, (a) wound healing, (b) Transwell migration, and (c) invasion analyses were performed as described in Materials and Methods. Data were represented as the mean ± SD of triplicate samples. Significant difference was observed from the control group (^*∗*^*p* < 0.05).

To further investigate the role of EGFR signaling pathway in propolin C-suppressed lung cancer cell migration and invasion, low endogenous EGFR-expressive A549 cells was chosen as study model. A549 lung cancer cells were pretreated with serial dosages of propolin C (2.5, 5, 7.5, and 10 *μ*M) for 30 min and then stimulated with 50 ng/mL of EGF for 24 h. After EGFR treatment, wound healing assay and* in vitro* cell migration and invasion assay were performed as described in Materials and Methods. As shown in Figure 5, the wound healing ability was induced after EGF stimulation (Figure 5(a)). EGF-induced wound healing ability was repressed in a dose-dependent manner in the cells pretreated with propolin C (Figure 5(a)). Meanwhile, EGF-induced* in vitro* migration and invasion activities were dose-dependently inhibited in A549 lung cancer cells pretreated with propolin C.

### 3.6. The Inhibition Mechanism of EGF-Induced EMT by Propolin C

**Figure 6:**
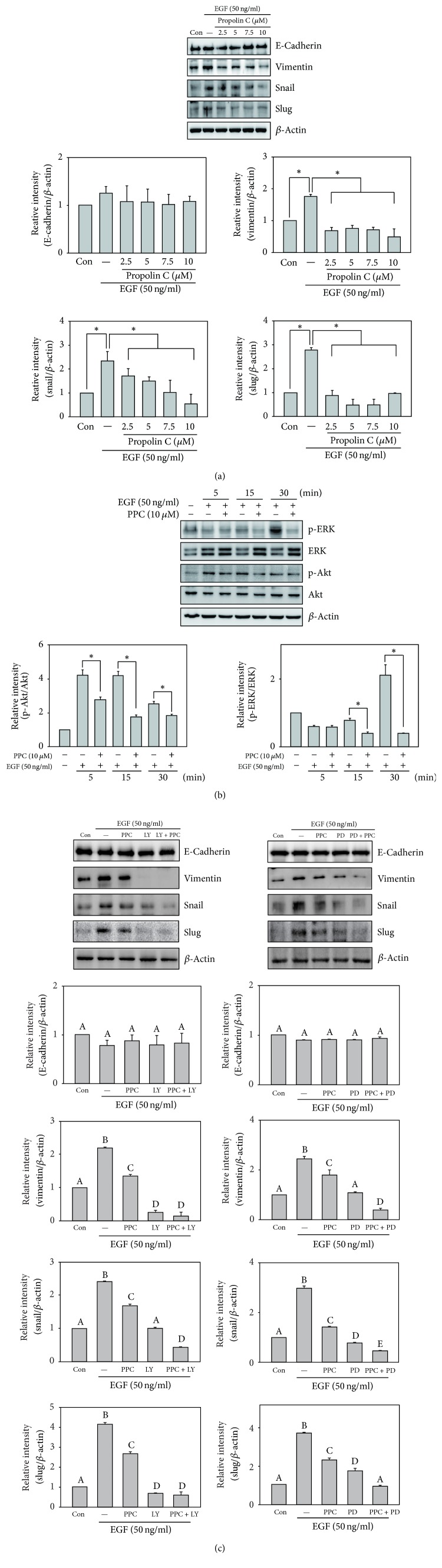
*Degeneration of EGF-induced EMT via PI3K/Akt and ERK inhibition in propolin C-treated A549 lung cancer cells*. A549 cells were synchronized and (a) pretreated with propolin C (2.5, 5, 7.5, and 10 *μ*M) or (b) pretreated with 10 *μ*M of propolin C (PPC) for 30 min and then stimulated with 50 ng/mL EGF for 5, 15, and 30 min. After EGF incubation, cell lysates were harvested and Western blot analyses were then performed to detect the expressions of E-cadherin, vimentin, slug, snail, phospho-ERK, ERK, phospho-Akt, Akt, and *β*-actin. Significant difference was observed from the compared groups (^*∗*^*p* < 0.05). (c) HCC827 cells were pretreated with propolin C (PPC, 10 *μ*M), LY294002 (LY, 10 *μ*M), and PD98059 (PD, 10 *μ*M) alone or cotreated with LY294002 or PD98059 with propolin C for 30 min and then incubated with EGF (50 ng/mL) for 24 h. After incubation, cells were harvested and Western blot analyses were used to detect E-cadherin, vimentin, snail, slug, and *β*-actin expressions. Data were shown as mean ± SD (*n* = 3). Different uppercase letters (A−D) indicate statistical differences among group (*p* < 0.05), and the same letter showed no difference (*p* > 0.05).

To demonstrate the inhibition mechanism of propolin C in EGF-induced lung cancer cell migration and invasion, EMT molecules expressions were evaluated. A549 lung cancer cells were pretreated with serial dosages of propolin C for 30 min and then incubated with EGF for 24 h. After EGF treatment, EMT molecule expressions were examined by Western blot analyses. Neither downregulation of E-cadherin expression was observed with EGF treatment nor upregulation of E-cadherin expression was measured in the pretreatment with propolin C compared with the control (Figure 6(a)). Interestingly, mesenchymal-like markers were only detected in the expression of EGF-regulated EMT. The expressions of vimentin, snail, and slug were upregulated in EGF treatment. Furthermore, EGF-induced expressions of vimentin, snail, and slug were inhibited in the pretreatment with propolin C.

The results revealed that propolin C-regulated EMT molecule expressions might be through downregulation of PI3K/Akt and ERK signaling pathways in EGFR-mutated HCC827 lung cancer cells (Figure 3). To verify the effects of propolin C in EGF/EGFR signaling pathway, the downstream EGF/EGFR signaling effectors, PI3K/Akt and ERK, were addressed. Increase of phospho-ERK expression was detected after EGF treatment for 15 min and the expression dramatically increased after 30 min of EFR treatment. However, EGF-induced ERK phosphorylation was inhibited by 10*μ*M of propolin C (Figure 6(b)). In PI3K/Akt signaling pathway, increasing expression of phospho-Akt was shown after 5 min of EGF treatment and it persisted to 30 min. EGF-induced Akt phosphorylation was repressed by the pretreatment with propolin C (Figure 6(b)). Subsequently, the biological roles of PI3K/Akt and ERK signaling pathways in propolin C-repressed EGF-induced EMT were examined. A549 lung cancer cells were pretreated with propolin C, LY294002, and PD98059 alone, respectively, or were cotreated with LY294002 and PD98059 with propolin C for 30 min and then stimulated with EGF for 24 h. The expressions of EMT molecules were then addressed by Western blot. As shown in Figure 6(c), significant difference of EGF-regulated E-cadherin expression was not perceived in the pretreatments with the inhibitors alone or cotreatment with propolin C and inhibitors. However, downregulation of EGF-induced vimentin, snail, and slug expressions was discovered in LY294002- or PD98059-treated cells. Furthermore, repressing EGF-induced mesenchymal-like molecule expression via propolin C was enhanced in cotreatment with LY294002 or PD98059 (Figure 6(c)).

## 4. Discussion

Searching and characterizing phytochemicals and exploring biological activities from foods or plant sources have become the prominent strategy for cancer chemoprevention [33]. Propolins belong to the family of c-prenylflavanones and 8 related compounds have been identified from Taiwanese propolis [13]. Although propolins have been demonstrated to possess anticancer activities in numerous cancer cell models, the antimigration and anti-invasion activities of propolins are still unclear. In present studies, the anti-migration and anti-invasion activities of propolin C in EGF/EGFR-mediated EMT in lung cancer cells were examined. The results revealed that inhibition of migration and invasion by propolin C was through downregulation of EGFR/PI3K/Akt and ERK-mediated EMT signaling pathways in EGFR-mutated HCC827 lung cancer cells. Moreover, reversing EGF-induced migration and invasion and EMT changes were observed in propolin C-treated EGFR wild-type A549 lung cancer cells. Activation of EGFR/PI3K/Akt and ERK signaling pathway by EGF was repressed via propolin C.

Malignant neoplasm metastasis is highly correlated with majority of deaths in cancer patients. Changing cells from immobile epithelial phenotype to more invasive mesenchymal phenotype via EMT is critical process for tumor metastasis [26]. During EMT progression, the expression of E-cadherin, the major epithelial-like molecule, is decreased concomitantly with the increase of mesenchymal-like molecules expressions [25]. Numerous studies indicate the high correlation between EMT and prognosis of cancer patients. Downregulation of E-cadherin expression in colon cancer patients is associated with high Tumor-Node-Metastasis (TNM) stage and distant metastasis [34]. Gene overexpression of mesenchymal-like markers and repressive expression of E-cadherin are indicated to reduce recurrence of free survival in breast cancer and NSCLC patients [35, 36]. Therefore, inhibiting EMT progression may benefit cancer patients at the risk of developing metastasis. In the present studies, the inhibitory effects on migration and invasion by propolin C, an active biological component from Taiwanese propolis, were investigated in EMT-regulated lung cancer cells. The results revealed that propolin C did not affect cell proliferation and cell cycle distribution in EGFR-mutated HCC827 cells under treatment with 10 *μ*M of propolin C (Figure 1). Treatment of propolin C with noncytotoxic effect suppressed HCC827 cell migration and invasion in a dose-dependent manner by wound healing and* in vitro* migration and invasion assay (Figure 2). Furthermore, Western blots showed that the expressions of vimentin and snail were dose-dependently decreased by propolin C. Inductive expression of E-cadherin was observed after treatment with 7.5 *μ*M of propolin C. However, significant difference in slug expression in propolin C-treated cells was not detected (Figure 3(a)). To confirm the molecular mechanism of propolin C in EMT regulation, PI3K/Akt and ERK signaling pathways were inspected. As shown in Figure 3(b), the expressions of phospho-ERK and phospho-Akt were repressed in a dose-dependent manner (Figure 3(b)). Increase of E-cadherin expression and repression of vimentin and snail expression were observed by pharmacological inhibitor of PI3K/Akt (LY294002) and ERK (PD98059). Interestingly, the enhancement of expression of E-cadherin was perceived in combinational treatment with PI3K/Akt or ERK inhibitor plus propolin C, whereas the expressions of vimentin and snail were dramatically decreased (Figure 3(c)). These results revealed that PI3K/Akt- and ERK-mediated EMT was inhibited by propolin C followed by migration and invasion suppression in HCC827 cells.

EGFR has been indicated to regulate cell proliferation, survival, and metastasis [22]. Genetic mutation of EGFR has been found in numerous cancers and indicated high correlation with poor prognosis, especially in lung cancers [21, 22]. Clinical study indicates that patients with EGFR mutation possess higher invasive activity than those harboring wild-type EGFR [37]. In addition, higher concentration of EGF in serum has been observed in lung cancer patients compared to healthy group [38]. Accordingly, blockade of EGF/EGFR-mediated migration and invasion might improve prognosis. Numerous studies indicate that EMT-mediated migration and invasion are regulated by EGF/EGFR signaling pathway [29, 30, 39]. In the present studies, the results unveiled that the expression of phospho-EGFR was dose-dependently inhibited by propolin C in EGFR-mutated HCC827 lung cancer cells (Figure 4(a)). To further validate the character of EGFR signaling pathway in propolin C-regulated EMT, EGFR tyrosine kinase inhibitor, ZD 1839, was selected. As shown in Figure 4(b), the expression of E-cadherin was increased and the expressions of vimentin and snail were decreased in ZD-treated HCC827 cells. Interestingly, the profiles of ZD-regulated EMT molecule expression were similar to propolin C-regulated experiment. The results implicated that inhibition of EMT-regulated cell migration and invasion via propolin C might be over EGFR signaling repression.

To further prove migration and invasion of EGFR-mediated EMT in propolin C-suppressed lung cancer cells, EGF-induced EGFR wild-type A549 lung cancer cell model was examined. The results showed that the migration and invasion abilities were increased in EGF-stimulated cells by wound healing and* in vitro* migration and invasion analyses. EGF-induced migration and invasion were dose-dependently suppressed in A549 cells pretreated with propolin C (Figure 5). Furthermore, the expressions of mesenchymal-like molecules, vimentin, snail, and slug, were upregulated after EGF stimulation, while expressions of EGF-induced mesenchymal-like molecules were inhibited via propolin C. Nevertheless, there was no significant difference in the expression of epithelial-like molecule, E-cadherin, in EGF-stimulated A549 cells with or without propolin C pretreatment (Figure 6(a)). In addition, EGF-induced expressions of vimentin, snail, and slug were suppressed by PI3K/Akt and ERK inhibitors alone or plus propolin C. Remarkably, EGF-inhibited E-cadherin expression was not upregulated after PI3K/Akt and ERK inhibitors alone or plus propolin C (Figure 6(c)). Recent studies have shown that the expression of E-cadherin is not increased after 24 h of EGF treatment in A549 cells [40]. However, downregulation of E-cadherin expression is noticed after EGF stimulation for 72 h in MCF-7 cells [29]. A long-term effect of downregulation of E-cadherin expression via EGF stimulation is suggested in our system. 24-Hour exposure might not be enough to detect the changes of E-cadherin expression in our system. Furthermore, our results also revealed that the expression of slug did not change after propolin C treatment in HCC827 cell (Figure 3(a)) but decreased in propolin C-treated A549 cells after EGF stimulation (Figure 6(c)). Not only is the expression of slug regulated by EGFR signaling pathway but also it is induced by hepatocyte growth factor (HGF)/c-Met-mediated signaling pathway in murine colorectal and lung cancer cells [41, 42]. Analyses of the endogenous expression of c-Met showed that higher expression of c-Met signaling pathway was observed in HCC827 than in A549 lung cancer cells [42]. We speculated that high expression of c-Met signaling pathway might bypass propolin C-inhibited slug expression in HCC827 lung cancer cells.

**Figure 7:**
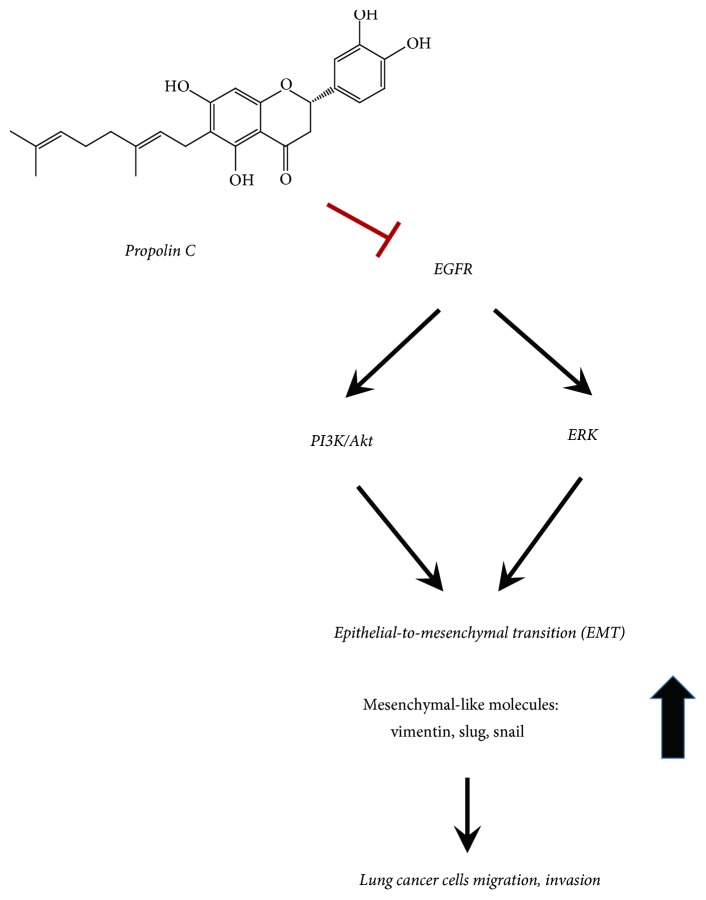
Proposed signal transduction pathways of the migration and invasion by propolin C in lung cancer cells.

Propolis has been indicated to possess beneficial effects on health and disease prevention. More than 300 biological components of propolis have been characterized from different countries or locations. The vast variations of these active components depend on the geographic regions, seasons, and plant sources [5–8, 12]. Propolins, which belong to c-prenylflavanones of Taiwanese propolis, have been identified and the biological activities have been characterized. The anticancer activities of propolins have been demonstrated in various cancer cell lines. Induction of mitochondria-dependent apoptosis by propolin A, B, and C has been observed in human melanoma cells [14, 15]. The highly radical scavenging activity of propolin G has been demonstrated to protect oxidative stress-induced cortical neuron damage [17]. Accumulation of G1 phase cells through p53-dependent and -independent p21^Waf1/Cip1^ expression via propolin H is also indicated in lung cancer cells [16]. Although the antitumorigenic activities of propolins have been examined, other biological activities of propolins are still unclear. In these studies, antimigration and anti-invasion activities of propolin C through EMT regulation were discovered. Furthermore, repression of EMT-regulated migration and invasion was through downregulation of EGFR-mediated PI3K/Akt and ERK signaling pathways in EGFR-mutated HCC827 lung cancer cells (Figures 3, 4, and 5). Additionally, EGF-induced PI3K/Akt and ERK activation was inhibited by propolin C in EGFR wild-type A549 lung cancer cells (Figure 6(b)). Accordingly, propolin C might be an inhibitor of EGFR. Although the results revealed that propolin C-regulated EMT might be through EGFR signaling pathway downregulation, other signaling pathways involved in EMT regulation could not be excluded. Many signaling pathways have also been indicated to regulate EMT, such as hepatocyte growth factor (HGF)/c-Met, transforming growth factor-beta 1 (TGF-*β*1), and fibroblast growth factor (FGF) signaling pathways [42–44]. The roles of propolin C in these signaling pathways-mediated migration and invasion should be further explored. The antitumorigenesis of propolin C in other signaling pathways is also investigated in our future study. In conclusion, inhibition of migration and invasion via propolin C was suggested by the inhibition of EGFR-mediated signaling pathway in lung cancer cells (Figure 7). Propolin C was suggested as an antitumorigenic candidate compound for lung cancer treatment and/or prevention.

## 5. Conclusions

The present results revealed that suppression of lung cancer cells migration and invasion with propolin C treatment was through EMT regulation. Propolin C-regulated EMT was through downregulation of EGFR-mediated PI3K/Akt and ERK signaling pathways in EGFR-mutated lung cancer cells. In addition, propolin C also inhibited EGF-induced migration and invasion as well as mesenchymal-like markers expressions in EGFR wild-type lung cancer cells. EGF-induced PI3K/Akt and ERK activation was inhibited in propolin C-repressed EMT in EGFR wild-type lung cancer cells. Taiwanese propolis active component, propolin C, might become an antitumorigenic candidate compound for lung cancer treatment and/or prevention in the future.
